# Associations of *ChREBP* and Global DNA Methylation with Genetic and Environmental Factors in Chinese Healthy Adults

**DOI:** 10.1371/journal.pone.0157128

**Published:** 2016-06-09

**Authors:** Jiajia Gao, Xueping Qiu, Xuebin Wang, Chunyan Peng, Fang Zheng

**Affiliations:** Center for Gene Diagnosis, Zhongnan Hospital of Wuhan University, Wuhan, China; University of Lleida, SPAIN

## Abstract

Age, gender, diet, gene and lifestyle have been reported to affect metabolic status and disease susceptibility through epigenetic pathway. But it remains indistinct that which factors account for certain epigenetic modifications. Our aim was to identify the influencing factors on inter-individual DNA methylation variations of *carbohydrate response element binding protein (ChREBP)* and global genome in peripheral blood leucocytes (PBLs). *ChREBP* DNA methylation was determined by bisulfite sequencing, and genomic 5mdC contents were quantified by capillary hydrophilic-interaction liquid chromatography/ in-source fragmentation/ tandem mass spectrometry system in about 300 healthy individuals. Eleven single nucleotide polymorphisms (SNPs) spanning *ChREBP* and *DNA methyltransferase 1* (*DNMT1)* were genotyped by high resolution melting or PCR-restriction fragment length polymorphism. *DNMT1* mRNA expression was analyzed by quantitative PCR. We found *ChREBP* DNA methylation levels were statistically associated with age (Beta (B) = 0.028, *p* = 0.006) and serum total cholesterol concentrations (TC) (B = 0.815, *p* = 0.010), independent of sex, concentrations of triglyceride, high density lipoprotein cholesterol, low density lipoprotein cholesterol (LDL-C), fasting blood glucose and systolic blood pressure, diastolic blood pressure, PBLs counts and classifications. The *DNMT1* haplotypes were related to *ChREBP* (odds ratio (OR) = 0.668, *p* = 0.029) and global (OR = 0.450, *p* = 0.015) DNA methylation as well as LDL-C, but not *DNMT1* expression. However, only the relation to LDL-C was robust to correction for multiple testing (OR_*FDR*_ = 1.593, *p*_*FDR*_ = 0.013). These results indicated that the age and TC were independent influential factors of *ChREBP* methylation and *DNMT1* variants could probably influence LDL-C to further modify *ChREBP* DNA methylation. Certainly, sequential comprehensive analysis of the interactions between genetic variants and blood lipid levels on *ChREBP* and global DNA methylation was required.

## Introduction

DNA methylation is a main epigenetic mechanism that affects gene transcription [1], tissue differentiation [2] and chromatin remodeling [3]. It has been reported that DNA methylation variations are involved in changes of the metabolic status [4–6], while the dietary component could also act as an epigenetic regulation agent against disease [7–11]. However, the underlying mechanisms of how environment or nutrition mediates through epigenetic pathway affecting disease susceptibility are still not clearly understood [12, 13]. These epigenetic modifications are likely to adjust expressions of important genes mediating pathophysiology processes, and are linked with direct benefits of diet and lifestyle, and might offer a rational and simple way to prevent diseases. In fact, investigations have implicated inter-individual DNA methylation variations with age, gender, diet, lifestyle, and genetic variants [14–18] especially single nucleotide polymorphisms (SNPs) in the DNA methyltransferases 1 (DNMT1), which could bind methyl groups to hemi-methylated DNA [19]. These SNPs could affect DNMT1 protein folding, catalytic activity and heterochromatin binding ability, thus leading to the changes of global and loci-specific DNA methylation [20–22]. But substantially less is known about the exact interactions among epigenetic variations, genetic variants and environmental factors.

ChREBP (GenBank accession number: NC_000007.14), is a transcription factor binding with genes of glucose, lipid and redox metabolism, and SNPs in *ChREBP* gene were reported to be associated with plasma triglyceride levels and coronary artery disease (CAD) in our previous study [23]. Furthermore, we found a distinct inter-individual DNA methylation variation in CpG island of C*hREBP* in peripheral blood leukocytes (PBLs). Then we speculate either or both of metabolite and heredity would lead to epigenetic modifications in *ChREBP*. Lipid and glucose levels and blood pressures were chosen as candidate influence factors based on ChREBP’s functions, and SNPs in *ChREBP* and *DNMT1* genes were selected as potential genetic cis-acting elements and trans-acting factors.

In order to reveal the modification factors on methylation variations in C*hREBP*, we investigated associations among the DNA methylation status of *ChREBP* gene plus global genome, genetic variations within *ChREBP* and *DNMT1* genes, the metabolite such as blood lipid levels and fasting blood glucose (FBG) etc.

## Materials and Methods

### Study population

The study population consisted of 309 healthy individuals recruited in Zhongnan hospital (Wuhan, China). General health was established using a general medical checklist. All subjects were free of medication and showed no signs of CAD, hypertension, diabetes mellitus or dyslipidemia based on the physical examination results at the time of enrollment. Informed consent was obtained from all subjects prior to their participation in the study from March/30/2012 to February/25/ 2014. Each subject’s clinical data and blood sample were collected and analyzed anonymously. The authors didn’t have access to identifying information. This study was approved and recorded in Medical Ethics Committee of Zhongnan Hospital of Wuhan University and met the declaration of Helsinki.

### Clinical Data

The systolic blood pressure (SBP) and diastolic blood pressure (DBP) were measured using a standard mercury sphygmomanometer. The serum concentrations of fasting blood glucose (FBG), total glyceride (TG), total cholesterol (TC), low density lipoprotein cholesterol (LDL-C), and high density lipoprotein cholesterol (HDL-C) were determined using the AU5400 automatic biochemical analyzer (Beckman Coulter Co. Ltd). PBLs differential counts were analyzed using the LH750 hematology analyzer (Beckman Coulter Co. Ltd). These analyzers were employed in the Core Laboratory of Zhongnan Hospital using standard techniques.

### SNP Selection and Genotyping

**Fig 1:**
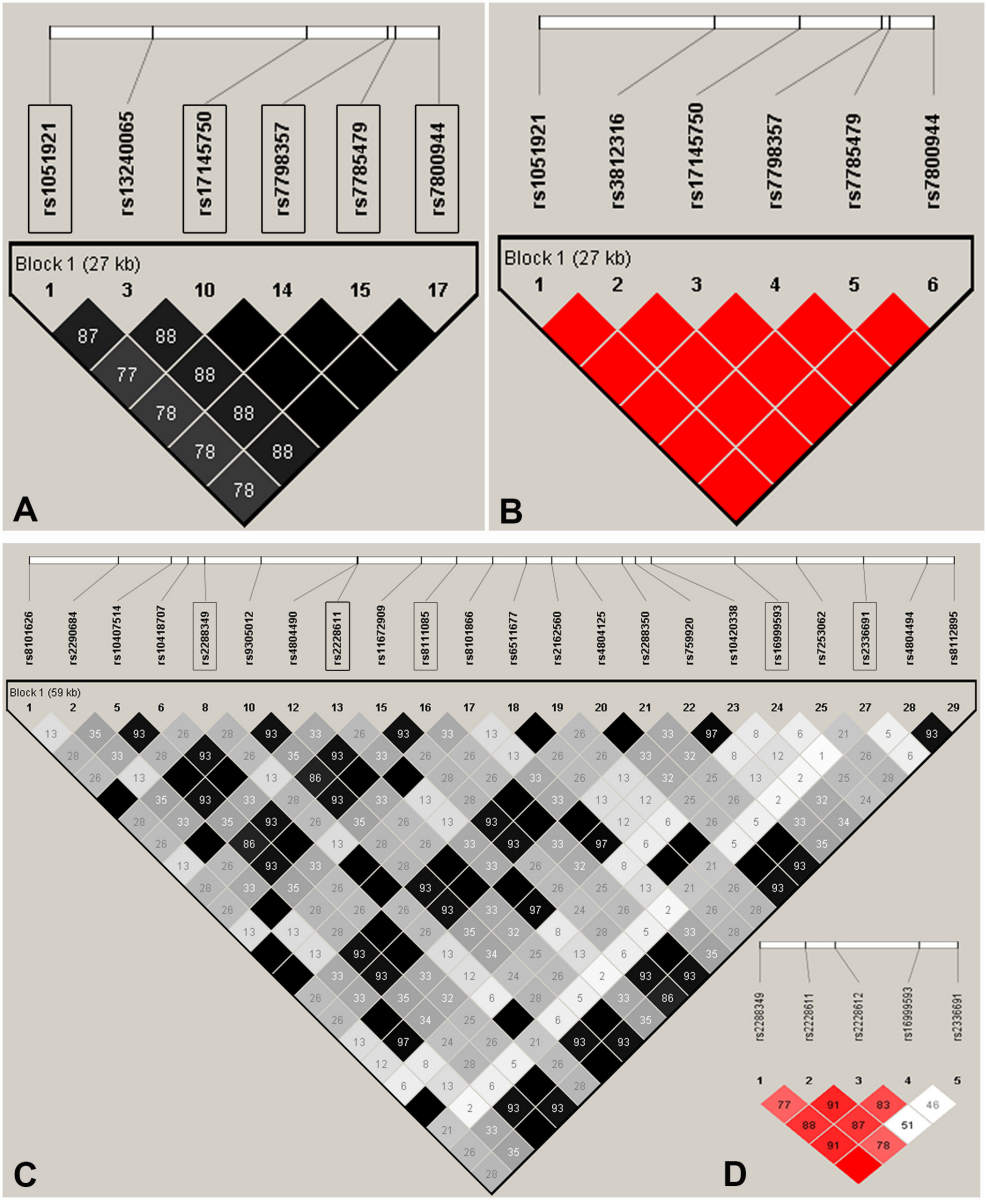
Linkage disequilibrium (LD)-plot. (**A**) LD-plot of *ChREBP* established using Haploview 4.2 program based on HapMap data. Five tag SNPs of *ChREBP* investigated in this research were highlighted in black boxes; (**B**) LD-plot of *ChREBP* in 50 individuals of our study population. The LD-plot was composed by 6 SNPs, including the 5 tag SNPs and 1 nonsynonymous SNP; (**C**) LD-plot of *DNMT1* established using Haploview 4.2 program based on HapMap data. Five tag SNPs in *DNMT1* investigated in this research were highlighted in black boxes; (**D**) LD-plot composed by the 5 tag SNPs of *DNMT1* in 287 individuals of our study population.

The SNPs were selected using Haploview 4.2 program [24] based on Chinese Han Beijing population (CHB) data from the HapMap database (http://www.hapmap.org, phase2). Two tag SNPs spanning *ChREBP* and five tag SNPs spanning *DNMT1* were chosen using pairwise r^2^ threshold of ≤ 0.8 and minor allele frequency (MAF) threshold of ≥ 0.05. The SNPs were rs1051921, rs17145750 within *ChREBP*, and rs2288349, rs2228611, rs8111085, rs16999593, rs2336691 within *DNMT1* (Fig 1A and 1C).

Any of 5 tag SNPs could be chosen as a gene tag in a block with high linkage disequilibrium (LD) pattern, from 1-kb region upstream to 1-kb downstream of *ChREBP*. And the high LD pattern of the 5 tag SNPs (rs1051921, rs17145750, rs7798357, rs7785479 and rs7800944) plus 1 nonsynonymous SNP (rs3812316) in *ChREBP* was confirmed in our study population (D` = 1, Fig 1B) [23]. On the other side, the picked 5 tag SNPs in *DNMT1* captured all 22 SNPs, from 1-kb region upstream to 1-kb downstream of *DNMT1* (GenBank accession number: NC_0000019.10). And 4 of the 5 tag SNPs of *DNMT1* (rs2288349, rs2228611, rs8111085 and rs16999593) were in linkage disequilibrium in our study population (D`≥ 0.77, Fig 1D) [25–27].

Genomic DNA of blood sample was isolated using standard proteinase K digestion and phenol-chloroform extraction. Nine SNPs were genotyped by high-resolution melting (HRM) on LightScanner 32 (Idaho Technology, USA). Two SNPs (rs3812316 & rs7798357) were genotyped by PCR-restriction fragment length polymorphism (PCR-RFLP) method due to G/C transversion. Ten percent of DNA samples were randomly selected for genotype verification using direct PCR sequencing (Qingke Company, Wuhan, China). The detail primer sequences are available in S1 and S2 Tables.

### Bisulfite sequencing for *ChREBP* DNA methylation

After spectrophotometric quantization, 2 ug of genomic DNA was treated with bisulfite as described previously [28]. Genomic DNA of PBLs was treated using CpG M.ssI methyltransferase (New England Biolabs) and was used as the methylated control, whereas the ‘C’ in non-CpG island (‘C’ completely transforming to ‘T’) was considered as the unmethylated control. Bisulfite DNA was amplified by PCR with bisulfite sequencing (BSP) primers designed by Primer 3.0 and listed in S3 Table. PCR products were cloned into the PMD18-T vector (Takara, Dalian, China), and ten positive clones from each sample were randomly selected for sequencing. DNA methylation levels were calculated by the percentage of methylated CpG sites divided by total CpG sites (290 CpG loci) in ten clones.

### LC-ESI-MS/MS analysis on genomic 5mdC contents

The capillary hydrophilic interaction chromatography (cHILIC) was performed on a Shimadzu Prominence nano-flow liquid chromatography system (Shimadzu, Tokyo, Japan) with two LC-20AD nano pumps, two vacuum degassers, a LC-20AB high performance liquid chromatography (HPLC) pump, a SIL-20AC HT auto-sampler, and a nano-flow control valve. The electrospray ionization/tandem mass spectrometry (ESI-MS/MS) experiment for detecting the genomic 5-mdC contents was detailly described in the previous study [29]. The results showed linearity within the range of 0.05% - 10% (molar ratio of 5-mdC/dC) with a coefficient value (R^2^) 0.996.

### Quantitative PCR of *DNMT1* expression

The first strand cDNA in PBLs was synthesized using RevertAid^™^ First Strand cDNA Synthesis Kit (Thermo Scientific lnc.) after mRNA was extracted by RNApure Blood Kit (CoWin Bioscience Co. Ltd). Quantitative PCR (qPCR) of *DNMT1* expression was performed in triplicate using iTaq^™^ Universal SYBR GREEN mix (BioRad) on a CFX96 Real-Time PCR Detection System (BioRad). The qPCR primer sequences were listed in S4 Table. The mRNA levels were normalized to *GAPDH*, and the results were expressed as mean ± standard deviation (SD).

### Statistical analysis

Continuous variables were expressed as mean ± SD or as median (interquartile range). The comparison of DNA methylation and expression levels among different genotypes was carried out using Mann-Whitney U test or Kruskal-Wallis H test. The correlations between DNA methylation and age, sex, blood pressure, blood index were analyzed by univariate regression and multivariate regression. LD and haplotype construction were analyzed by the Haploview4.2 and the SHEsis software platform (http://analysis.bio-x.cn/myAnalysis.php). The SHEs is a program that uses a partition ligation-combination-subdivision EM algorithm in haplotype reconstruction and frequency estimation. The associations were tested on most likely haplotypes [30, 31]. Data was analyzed with SPSS software (version 16.0) and a *p* value < 0.05 (two-tailed) was considered statistically significant. False Discovery Rate (FDR) was applied for multiple testing corrections. The *p*_FDR_ value was calculated by multiplying its *p* value by the number of tests performed and then divided by the rank order of each *p* value (where rank order 1 is assigned to the smallest *p* value). An FDR of 0.05 was used as a critical value to assess whether *p*_FDR_ value was significant [32].

## Results

### *ChREBP* DNA methylation was independently related to age and serum TC concentrations

A description of the study population is reported in Table 1.

**Table 1:** Clinical characteristics of the study population.

Clinical characteristic	N	Mean ± SD	Median (interquartile range)
age, years	309	55.1 ± 10	53 (47–62)
sex, male/female	139/170		
SBP, mmHg	309	116 ± 15	115 (106–126)
DBP, mmHg	309	70 ± 9	70 (63–76)
TC, mmol/L	309	4.34 ± 0.54	4.42 (4.03–4.70)
LDL-C, mmol/L	309	2.55 ± 0.36	2.61 (2.35–2.83)
HDL-C, mmol/L	309	1.32 ± 0.21	1.31 (1.18–1.43)
TG, mmol/L	309	0.92 ± 0.34	0.89 (0.69–1.13)
FBG, mmol/L	309	4.77 ± 0.45	4.73 (4.47–5.06)
PBLs counts, (×10^9^/L)	309	5.61 ± 1.37	5.42 (4.71–6.35)
GRAN counts, (×10^9^/L)	309	59.10 ± 8.02	59.60 (53.80–64.60)
LYM counts, (×10^9^/L)	309	33.91 ± 7.79	33.3 (28.4–39.05)
MONO counts, (×10^9^/L)	309	7.02 ± 2.59	7.20 (5.80–8.55)
*ChREBP* DNA methylation, %	309	21.05 ± 13.57	18.60 (11.03, 27.93)
global DNA methylation, %	159	4.41 ± 0.84	4.18 (3.75, 5.02)
DNMT1 expression	158	0.007 ± 0.0078	0.004 (0.0025, 0.0099)

SBP, systolic blood pressure; DBP, diastolic blood pressure; TC, total cholesterol concentrations; LDL-C, low density lipoprotein cholesterol concentrations; HDL-C, high density lipoprotein cholesterol concentrations; TG, total triglyceride concentrations; FBG, fasting blood glucose concentrations; PBLs, peripheral blood leukocytes; GRAN, granulocytes; LYM, lymphocytes; MONO, monocytes.

We found that *ChREBP* DNA methylation was correlated with age, TC, TG, LDL-C (all *p* < 0.05), but was not related to sex, HDL-C, FBG, SBP, DBP, PBLs counts and classifications (all *p* > 0.05). However, after forward stepwise multivariate linear regression, only age and TC were independent factors associated with *ChREBP* DNA methylation, explaining 6.9% variation in *ChREBP* DNA methylation (Table 2).

**Table 2:** Associations of clinical characteristics with *ChREBP* DNA methylation.

Clinical characteristics	*ChREBP* DNA methylation			
	Univariate association		Multivariate association	
	B	*p*	B	*p*
**age**	0.030	**0.000**	0.028	**0.006**
sex	-0.317	0.060	-	-
**TC**	0.427	**0.006**	0.815	**0.010**
**TG**	0.517	**0.038**	-	-
HDL-C	-0.408	0.304	-	-
**LDL-C**	0.550	**0.018**	-	-
FBG	-0.025	0.893	-	-
SBP	0.001	0.862	-	-
DBP	-0.018	0.053	-	-
PBLs counts	0.024	0.694	-	-
GRAN counts	-0.002	0.872	-	-
LYM counts	0.002	0.869	-	-
MONO counts	0.047	0.235	-	-
R^2^			0.069	

B, Beta; SBP, systolic blood pressure; DBP, diastolic blood pressure; TC, total cholesterol concentrations; LDL-C, low density lipoprotein cholesterol concentrations; HDL-C, high density lipoprotein cholesterol concentrations; TG, total triglyceride concentrations; FBG, fasting blood glucose concentrations; PBLs, peripheral blood leukocytes; GRAN, granulocytes; LYM, lymphocytes; MONO, monocytes. The levels of *ChREBP* DNA methylation was sqrt-transformed, *p* < 0.05 was considered statistically significant (in bold).

### Associations between *ChREBP* DNA methylation and *DNMT1* haplotype

Because the six SNPs in *ChREBP* in our study have constructed a high LD pattern (Fig 1B), only 2 SNPs (rs1051921, rs17145750) were chosen to represent the haplotype of *ChREBP*. However, we didn’t identify any significant association between individual SNP or haplotype and levels of *ChREBP* DNA methylation (S5 and S6 Tables).

Since *DNMT1* plays a major role in the maintenance of methylation patterns, 5 tag SNPs within *DNMT1* were genotyped to estimate the trans-effect of genetic variants on *ChREBP* DNA methylation. Though no significant association was observed between single *DNMT1* SNP and *ChREBP* DNA methylation (S5 Table), significant difference was found in the frequency of the GAAT haplotype of *DNMT1* (composed of rs2288349, rs2228611, rs8111085 and rs16999593), between the subgroups with differential levels of *ChREBP* DNA methylation (*p* = 0.029, OR = 0.668, 95% CI = 0.465–0.960, Table 3). But after FDR correction, no significant association was observed.

**Table 3:** Comparisons of *DNMT1* haplotype distributions in subgroups with the higher and lower levels of *ChREBP* DNA methylation.

*DNMT1* haplotype	Haplotype frequencies (N (ratio))		*p*	OR	95% CI	*p*_*FDR*_
	Group 1	Group 2				
AGAT	73 (0.255)	68 (0.238)	0.641	1.095	0.747–1.606	0.641
**GAAT**	75 (0.260)	99 (0.343)	**0.029**	0.668	0.465–0.960	0.145
GGAT	39 (0.136)	27 (0.092)	0.098	1.554	0.920–2.625	0.163
GGGC	40 (0.140)	49 (0.170)	0.321	0.794	0.504–1.253	0.401
GGGT	48 (0.167)	33 (0.115)	0.070	1.553	0.962–2.508	0.163

The population was divided into two subgroups with the lower and higher levels of *ChREBP* DNA methylation by the median level of 18.60%. Group 1 was composed of the individuals with the levels of *ChREBP* DNA methylation less than 18.60%; Group 2 was composed of the individuals with the levels of *ChREBP* DNA methylation more than or equal to 18.60%. Loci for the haplotype analysis: rs2288349, rs2228611, rs8111085, and rs16999593. N = 287; *p*_*FDR*_, the adjusted *p* for multiple testing; Bold letter indicates the *p* value < 0.05. (All those haplotype frequencies < 0.03 will be ignored in analysis.)

### Associations of *DNMT1* haplotype with global DNA methylation and *DNMT1* expression

To further verify the possible effect of the *DNMT1* haplotype on DNA methylation, the influence of the *DNMT1* haplotype on global DNA methylation was analyzed. We observed a significant difference in haplotype GGGT frequencies between subgroups with the higher and lower levels of global DNA methylation (*p* = 0.015, OR = 0.450, 95% CI = 0.234–0.863, Table 4) before FDR correction.

**Table 4:** Comparisons of *DNMT1* haplotype distributions in subgroups with the higher and lower levels of global DNA methylation.

*DNMT1* haplotype	Haplotype frequencies (N (ratio))		*P*	OR	95% CI	*p*_*FDR*_
	Group 3	Group 4				
AGAT	49(0.310)	35(0.217)	0.052	1.653	0.994–2.749	0.130
GAAT	43(0.270)	46(0.286)	0.798	0.937	0.572–1.537	0.909
GGAT	15(0.092)	16(0.097)	0.909	0.957	0.451–2.030	0.909
GGGC	29(0.185)	27(0.169)	0.666	1.136	0.637–2.027	0.909
**GGGT**	16(0.099)	32(0.198)	**0.015**	0.450	0.234–0.863	0.090
AGAT	20(0.126)	17(0.106)	0.553	1.232	0.618–2.455	0.909

The population was divided into two subgroups with the lower and higher levels global DNA methylation by the median level of 4.18%. Group 3 was composed of the individuals with the level of global DNA methylation less than 4.18%; Group 4 was composed of the individuals with the level of global DNA methylation more than or equal to 4.18%. Loci for the haplotype analysis: rs2288349, rs2228611, rs8111085, and rs16999593. N = 159; *p*_*FDR*_, the adjusted *p* for multiple testing; Bold letter indicates the *p* value < 0.05. (All those haplotype frequencies < 0.03 will be ignored in analysis.)

In order to reveal the mechanism underlying the possible relation of *DNMT1* haplotypes with *ChREBP*, and global DNA methylation, we speculated that the *DNMT1* haplotype may affect global and specific-locus DNA methylation through regulation on the mRNA expression level of *DNMT1*. Sequentially, the mRNA expression level of *DNMT1* was measured and we didn’t reveal any significant association between *DNMT1* haplotypes and expression (S7 Table). However we did find a statistical association between *DNMT1* haplotypes and LDL-C even after FDR correction (Table 5), though we only find 2 SNPs were associated with lipid levels before FDR correction (S8 Table).

**Table 5:** Comparisons of *DNMT1* haplotype distributions in subgroups with the higher and lower levels of serum LDL-C.

*DNMT1* haplotype	Haplotype frequencies (N (ratio))		*p*	OR	*p*_*FDR*_	OR_*FDR*_
	Group 5	Group 6				
AGAT	66 (0.228)	74 (0.262)	0.333	0.828	0.326	0.812
GAAT	92 (0.317)	83 (0.2920)	0.527	1.123	0.419	1.000
GGAT	34 (0.117)	31 (0.108)	0.691	1.111	0.644	0.994
**GGGC**	34 (0.117)	55 (0.195)	**0.008**	0.534	**0.010**	0.561
**GGGT**	52 (0.180)	28 (0.100)	**0.006**	1.985	**0.013**	1.593

The population was divided into two subgroups with the lower and higher levels of serum LDL-C by the median level of 2.62 mmol/L. Group 5 was composed of the individuals with serum LDL -C levels less than 2.62 mmol/L; Group 6 was composed of the individuals with serum LDL-C levels more than or equal to 2.62 mmol/L. Loci for the haplotype analysis: rs2288349, rs2228611, rs8111085, and rs16999593. N = 287; *p*_*FDR*_, the adjusted *p* for multiple testing; OR_*PDR*_, the adjusted OR for multiple test. Bold letter indicates the *p* value < 0.05. (All those haplotype frequencies < 0.03 will be ignored in analysis.)

## Discussion

In this study, we analyzed the DNA methylation of *ChREBP* and global genome in PBLs, using BSP and LC-ESI-MS/MS. We found that age and serum TC were independent modification factors of *ChREBP* DNA methylation, and observed an association related LDL-C to *DNMT1* haplotypes which have nominal relationships with the DNA methylation of *ChREBP* and global genome. As reported, the genetic and epigenetic mechanisms independently involved in the pathophysiological processes and disease developments [12, 13], however they might interact in some processes to determine disease susceptibility together. In our study, we presented a perspective on whether there were interactions between metabolites, genetic variants and epigenetic modifications of DNA methylation.

PBLs are good in vivo target cells for investigating the DNA methylation levels in *ChREBP* gene and global genome, because the peripheral blood was easy to be collected and assayed. Furthermore, as reported by Davies and Smith et al., the modification of DNA methylation status in PBLs could reflect the modification on DNA methylation in other organs [33, 34]. We found an association between *ChREBP* DNA methylation of PBLs and serum TC. It might indicate a negative feedback of down-regulation on *ChREBP* expression mediated by DNA methylation under cell microenvironments with higher serum lipid levels, since *ChREBP* could activate the transcription of lipid metabolism genes [35]. It also could be a reflection of the DNA methylation modification in liver induced by the elevated cholesterol level.

Bollati et al. also found a complex relationship among the DNA methylation of *tumor necrosis factor α (TNFα)* in PBLs and blood levels of LDL-C, TC/HDL-C and LDL-C/HDL-C [36]. And Gillberg et al. found the DNA methylation of *peroxisome proliferator activated receptor gamma coactivator 1 alpha (PPARGC1A)* in subcutaneous adipose tissue was influenced by high-fat overfeeding in a birth weight dependent manner [37].

Furthermore, we found that the higher level of *ChREBP* methylation was associated with aging, which was consistent with the previous literatures. Barbara et al. reported that *CALCA* and *MGMT* methylation levels increased with age in PBLs[38]. Tra et al. also confirmed that the DNA methylation level of 23 loci elevated with age in T lymphocytes [39], while Fuke et al. found that the genome methylation level decreased during the aging process in PBLs [14]. These results suggested there could be a contrary age-related alteration of DNA methylation between global genome and specific genes in PBLs.

Moreover, we observed that serum LDL-C were related to *DNMT1* haplotypes, while *ChREBP* and global DNA methylation were only nominally associated with *DNMT1* haplotypes. Actually we reported a risk association of *DNMT1* SNP rs2228611 with CAD in Han population, but we didn’t investigate the relation between SNPs and lipid levels in this previous study [40]. The influence of *DNMT1* haplotype on LDL-C might be the underlying reason involving *DNMT1* SNPs with CAD, and probably ascribed to DNMT1 functions on DNA methylation of specific lipid metabolism genes. The association of LDL-C with the interaction among SNPs as haplotyps, but not a single SNP, was similar to some other studies [41–43]. And several investigations have reported the influence of *DNMT1* haplotypes on specific loci. Potter et al. reported associations of both maternal and infant *DNMT3B* genotypes with *IGFBP3* methylation levels in the infants [44]. Boks et al. identified associations of genetically heritable SNPs with differences in DNA methylation levels not in the same chromosome, which is similar to the trans-effects of the *DNMT1* haplotype on *ChREBP* methylation [45]. However, we didn’t find any statistically significant relationship between SNP and DNA methylation levels, which might be due to the limited sample size or other disturbances. Overall, compared to genetics variants, metabolites such as TC and the environmental factors such as age played a dominant role on epigenetics variations.

In addition, serum LDL-C was associated not only with the *DNMT1* haplotype (Table 2) but also with *ChREBP* DNA methylation (Table 5). Whether these indicated that genetic factors indirectly adjusted the *ChREBP* DNA methylation through influencing the metabolite concentration needed further investigation, and this hypothesis could probably explain associations between the *DNMT1* haplotype and *ChREBP* DNA methylation before FDR correction.

Our study has some limitations. Firstly, because we have investigated only 11 SNPs of *ChREBP* and *DNMT1* genes instead of global genome, we think that comprehensive studies would be more efficient for finding genetic variants affecting DNA methylation variations. Secondly, we didn’t investigate the functional mechanism for the association between *ChREBP* DNA methylation and serum TC. Thirdly, lipid concentrations could be influenced by other genetic and epigenetic variability, which might be the confusing factors in the association study between *DNMT1* haplotypes and lipid levels, and should be included for future researches.

In conclusion, this study explored the complex regulator network among metabolites and epigenetic and genetic variations. The results showed that age and serum TC were the modification factors on inter-individual variation of *ChREBP* DNA methylation, and genetic variants might indirectly influence *ChREBP* DNA methylation through adjusting metabolite blood levels. If metabolites could modify an individual’s epigenetic status, it would be a good fundament for diet therapy and a strong support for healthy lifestyle for the benefit of individuals and for the sake of offsprings. And in the future, we might find some way to amend the genetic code in an epigenetic way.

## Supporting Information

S1 Dataset Raw data of all indexes except DNMT1 expression. (XLSX)

S2 Dataset Raw data of *DNMT1* expression and SNP genotypes. (XLSX)

S1 Table Primers used for genotyping and DNA sequencing for *ChREBP* SNPs. (DOCX)

S2 Table Primers used for HRM and DNA sequencing for *DNMT1* SNPs. (DOCX)

S3 Table Primers used for *ChREBP* bisulfite sequencing. (DOCX)

S4 Table Primers used for DNMT1 mRNA expression analysis. (DOCX)

S5 Table Associations of SNPs in *ChREBP* and *DNMT1* with *ChREBP* and global DNA methylation and *DNMT1* expression. (DOCX)

S6 Table Comparisons of *ChREBP* haplotype distributions in subgroups with the higher and lower levels of *ChREBP* DNA methylation. (DOCX)

S7 Table Comparisons of *DNMT1* haplotype distributions in subgroups with the higher and lower levels of *DNMT1* mRNA expression. (DOCX)

S8 Table Associations between *DNMT1* SNPs and lipid levels. (DOCX)
