# Supplementary material for: Associations of ChREBP and Global DNA Methylation with Genetic and Environmental Factors in Chinese Healthy Adults
Source: PLoS One. 2016 Jun 9;11(6):e0157128. doi: 10.1371/journal.pone.0157128 (PMC4900669; doi:10.1371/journal.pone.0157128)
Supplement: S1 Table — (DOCX) [file pone.0157128.s003.docx]

S1 Table. Primers used for genotyping and DNA sequencing for *ChREBP* SNPs.

| *ChREBP* SNPs | |  | Primers (5’ →3’) | Amplicon length |
| --- | --- | --- | --- | --- |
| rs1051921 | HRM | Forward: | TGACCTTGGGTGACTCG | 59bp |
|  |  | Reverse: | CCCATTTTGCAGATTGAAACACA |  |
|  | DNA | Forward: | GGGTCCAGGTTTCACAC | 245bp |
|  | sequencing | Reverse: | CTCCTCTTTCCACCGTTG |  |
| rs17145750 | HRM | Forward: | ACACCAGCAAAGATGAAGTT | 55bp |
|  |  | Reverse: | GCTACAGGCGTGAGCCA |  |
|  | DNA | Forward: | AAACAAAGGGCTGGGTCC | 454bp |
|  | sequencing | Reverse: | GCGCCTGGCCTAACAAAC |  |
| rs7785479 | HRM | Forward: | GGTGGGTGCCAGTCTGA | 59bp |
|  |  | Reverse: | GGCCCAATGACCAATGC |  |
|  | DNA | Forward: | CCCTGAGTAGCTGGGATT | 429bp |
|  | sequencing | Reverse: | GCTGGGCTGTGTATCTTATT |  |
| rs7800944 | HRM | Forward: | ATATAGTCACTCCCCTGGGACA | 51bp |
|  |  | Reverse: | CTGGAGGGGAGCAGATTAC |  |
|  | DNA | Forward: | GTTGGCCAGGCTGGTCT | 323bp |
|  | sequencing | Reverse: | AATGAGGCCTGTTGTCCCTG |  |
| rs3812316 | RFLP and | Forward: | CCAGCCATCCCTCCCTT | 500bp |
|  | sequencing | Reverse: | CCTGTATCTGCATCCTGGTCTAT |  |
| rs7798357 | RFLP and | Forward: | GAGGCGAGGTCAGAGGAT | 273bp |
|  | sequencing | Reverse: | CTGGCTTGTGGATGGAGA |  |
