# Supplementary material for: Associations of ChREBP and Global DNA Methylation with Genetic and Environmental Factors in Chinese Healthy Adults
Source: PLoS One. 2016 Jun 9;11(6):e0157128. doi: 10.1371/journal.pone.0157128 (PMC4900669; doi:10.1371/journal.pone.0157128)
Supplement: S2 Table — (DOCX) [file pone.0157128.s004.docx]

S2 Table. Primers used for HRM and DNA sequencing for *DNMT1* SNPs.

| *DNMT1* SNPs | |  | Primers (5’ →3’) | Amplicon length |
| --- | --- | --- | --- | --- |
| rs2288349 | HRM | Forward: | ACAGAAACATAAGGCCCTGAG | 56bp |
|  |  | Reverse: | AGCTGGCAGTAGCTGCTGC |  |
|  | DNA | Forward: | TAGTAGAGGACCCGGCTAT | 299bp |
|  | sequencing | Reverse: | TGTTCTCTCTGGGCTTTGG |  |
| rs2228611 | HRM | Forward: | AGCAAAACCAATCTATGATGA | 56bp |
|  |  | Reverse: | TCCCGGACTATTCCTTACC |  |
|  | DNA | Forward: | CTGATCTGAAGTCTGCACGA | 260bp |
|  | sequencing | Reverse: | CAGATGTGAGCCACCCT |  |
| rs8111085 | HRM | Forward: | ACCTGAAAAAGTAAATCCACA | 60bp |
|  |  | Reverse: | ACCTTTACCTTTTCATCCT |  |
|  | DNA | Forward: | GGTTAGTGTTTCTAAGCTGCTAC | 204bp |
|  | sequencing | Reverse: | GCAACATGGCGAAACCC |  |
| rs16999593 | HRM | Forward: | AGACGTCCATTCACTTCCC | 55bp |
|  |  | Reverse: | ATTTGTCCTTGGAGAACGG |  |
|  | DNA | Forward: | CTGTTGGCATCTGCCATT | 254bp |
|  | sequencing | Reverse: | ATGATGATGATGATGATGATGAAGG |  |
| rs2336691 | HRM | Forward: | CCAATCTAATCACTTCAGCCC | 56bp |
|  |  | Reverse: | CGCTCTCTGCAGCTAGAAA |  |
|  | DNA | Forward: | GGGCCCAATCTAATCACTTCA | 300bp |
|  | sequencing | Reverse: | GTGTAAGCAGGCTGCATTC |  |
