# Supplementary material for: Associations of ChREBP and Global DNA Methylation with Genetic and Environmental Factors in Chinese Healthy Adults
Source: PLoS One. 2016 Jun 9;11(6):e0157128. doi: 10.1371/journal.pone.0157128 (PMC4900669; doi:10.1371/journal.pone.0157128)
Supplement: S3 Table — (DOCX) [file pone.0157128.s005.docx]

S3 Table. Primers used for *ChREBP* bisulfite sequencing.

| Gene |  | Primers (5’ →3’) | Amplicon length |
| --- | --- | --- | --- |
| *ChREBP*-W | Forward: | TTTTTGGAGTAAAGTAGGGG | 558bp |
|  | Reverse: | CTATAAACCAAACTCAAACACTC |  |
| *ChREBP*-N | Forward: | ATGAGGTTCGGTTGGTTAAGAGT | 248bp |
|  | Reverse: | TACGACGACGACACCATAAAATA |  |
