# Supplementary material for: Associations of ChREBP and Global DNA Methylation with Genetic and Environmental Factors in Chinese Healthy Adults
Source: PLoS One. 2016 Jun 9;11(6):e0157128. doi: 10.1371/journal.pone.0157128 (PMC4900669; doi:10.1371/journal.pone.0157128)
Supplement: S4 Table — (DOCX) [file pone.0157128.s006.docx]

S4 Table. Primers used for *DNMT1* mRNA expression analysis.

| Gene |  | Primers (5’ →3’) | Amplicon length |
| --- | --- | --- | --- |
| *GAPDH* | Forward: | GAAGGTGAAGGTCGGAGTC | 558bp |
|  | Reverse: | GAAGATGGTGATGGGATTTC |  |
| *DNMT1* | Forward: | ACCGCTTCTACTTCCTCGAGGCCTA | 248bp |
|  | Reverse: | GTTGCAGTCCTCTGTGAACACTGTGG |  |
