# Supplementary material for: Associations of ChREBP and Global DNA Methylation with Genetic and Environmental Factors in Chinese Healthy Adults
Source: PLoS One. 2016 Jun 9;11(6):e0157128. doi: 10.1371/journal.pone.0157128 (PMC4900669; doi:10.1371/journal.pone.0157128)
Supplement: S5 Table — (DOCX) [file pone.0157128.s007.docx]

S5 Table. Associations of SNPs in *ChREBP* and *DNMT1* with *ChREBP* and global DNA methylation and *DNMT1* expression.

|  |  | *ChREBP* methylation | | | | | |  |  |
| --- | --- | --- | --- | --- | --- | --- | --- | --- | --- |
| SNP |  | AA | | Aa | | aa | | Additive |  |
|  | Alleles | N | Median  (interquartile range) | N | Median  (interquartile range) | N | Median  (interquartile range) | z | *p* |
| *ChREBP* rs1051921 | C/T | 155 | 19.31 (12.41 - 28.97) | 40 | 17.75 (11.21 - 25.60) |  |  | -1.233 | 0.217 |
| *ChREBP* rs17145750 | C/T | 159 | 19.31 (12.41 - 28.97) | 36 | 17.42 (10.43 - 24.66) |  |  | -0.666 | 0.506 |
| *DNMT1* rs2288349 | G/A | 152 | 18.11 (10.70 - 28.97) | 117 | 18.97 (11.73 - 26.38) | 18 | 19.83 (14.31 - 27.25) | 0.056 | 0.973 |
| *DNMT1* rs2228611 | G/A | 129 | 18.97 (12.76 - 27.94) | 127 | 18.62 (11.03 - 28.97) | 31 | 15.17 (8.28 - 26.55) | 3.044 | 0.218 |
| *DNMT1* rs8111085 | A/G | 138 | 18.44 (10.60 - 28.45) | 122 | 19.31 (10.95 - 28.37) | 27 | 18.62 (12.76 - 24.83) | 0.390 | 0.823 |
| *DNMT1* rs16999593 | T/C | 194 | 20.00 (10.96 - 29.83) | 85 | 17.24 (10.00 - 24.48) | 8 | 19.32 (14.40 - 24.99) | 3.492 | 0.174 |
| *DNMT1* rs2336691 | G/A | 229 | 18.62 (10.73 - 28.28) | 58 | 19.14 (12.50 - 27.07) |  |  | -0.118 | 0.906 |
|  |  | Global methylation | | | | | |  |  |
|  |  | AA | | Aa | | aa | |  |  |
|  |  | N | Median  (interquartile range) | N | Median (interquartile range) | N | Median  (interquartile range) |  |  |
| *DNMT1* rs2288349 | G/A | 79 | 4.14 (3.70 - 5.02) | 70 | 4.14 (3.77 - 5.02) | 10 | 4.73 (4.44 - 5.54) | 3.793 | 0.150 |
| *DNMT1* rs2228611 | G/A | 77 | 4.25 (3.70 - 4.86) | 68 | 4.12 (3.72 - 5.33) | 14 | 4.15 (3.79 - 4.93) | 0.093 | 0.954 |
| *DNMT1* rs8111085 | A/G | 66 | 4.52 (3.86 - 5.08) | 82 | 4.12 (3.65 - 4.92) | 11 | 3.92 (3.59 - 5.06) | 4.848 | 0.089 |
| *DNMT1* rs16999593 | T/C | 102 | 4.14 (3.72 - 5.12) | 52 | 4.26 (3.73 - 4.89) | 5 | 4.59 (4.23 - 5.13) | 1.017 | 0.601 |
| *DNMT1* rs2336691 | G/A | 120 | 4.15 (3.71 - 5.05) | 39 | 4.43 (3.89 - 4.74) |  |  | -0.456 | 0.648 |
|  |  | DNMT1 expression | | | | | |  |  |
|  | AA | | | Aa | | aa | |  |  |
|  |  | N | Median  (interquartile range) | N | Median  (interquartile range) | N | Median  (interquartile range) |  |  |
| *DNMT1* rs2288349 | G/A | 96 | 2.37 (1.99 - 2.62) | 56 | 2.43 (2.08 - 2.60) | 6 | 2.39 (2.29 - 2.48) | 0.021 | 0.989 |
| *DNMT1* rs2228611 | G/A | 92 | 2.33 (2.11 - 2.58) | 57 | 2.40 (1.95 - 2.64) | 9 | 2.42 (2.21 - 2.53) | 0.067 | 0.967 |
| *DNMT1* rs8111085 | A/G | 61 | 2.24 (1.94 - 2.56) | 76 | 2.40 (2.11 - 2.64) | 21 | 2.32 (2.03 - 2.58) | 1.107 | 0.575 |
| *DNMT1* rs16999593 | T/C | 103 | 2.40 (2.14 - 2.63) | 53 | 2.29 (1.95 - 2.58) | 2 | 2.12 (2.01 - 2.23) | 2.079 | 0.354 |
| *DNMT1* rs2336691 | G/A | 125 | 2.37 (2.00 - 2.60) | 33 | 2.31 (2.18 - 2.48) |  |  | -0.295 | 0.768 |
