# Supplementary material for: Associations of ChREBP and Global DNA Methylation with Genetic and Environmental Factors in Chinese Healthy Adults
Source: PLoS One. 2016 Jun 9;11(6):e0157128. doi: 10.1371/journal.pone.0157128 (PMC4900669; doi:10.1371/journal.pone.0157128)
Supplement: S6 Table — (DOCX) [file pone.0157128.s008.docx]

S6 Table. Comparisons of *ChREBP* haplotype distributions in subgroups with the higher and lower levels of *ChREBP* DNA methylation.

| *ChREBP* haplotype | Haplotype frequencies (N (ratio)) | | *p* | OR | 95% CI |
| --- | --- | --- | --- | --- | --- |
|  | Subgroup with Lower Levels of *ChREBP* DNA methylation | Subgroup with the Higher Levels of *ChREBP* DNA methylation |  |  |  |
| CC | 180 (0.918) | 169 (0.871) | 0.330 | 1.419 | 0.703- 2.862 |
| TT | 15 (0.077) | 20 (0.103) | 0.330 | 0.705 | 0.349- 1.422 |

The population was divided into two subgroups with the lower and higher levels of *ChREBP* DNA methylation by the median of 18.60%.

Loci for the haplotype analysis: rs1051921, rs17145750; N=195.

*p* > 0.05. (All those haplotype frequencies <0.03 will be ignored in analysis.)
