# Supplementary material for: Associations of ChREBP and Global DNA Methylation with Genetic and Environmental Factors in Chinese Healthy Adults
Source: PLoS One. 2016 Jun 9;11(6):e0157128. doi: 10.1371/journal.pone.0157128 (PMC4900669; doi:10.1371/journal.pone.0157128)
Supplement: S7 Table — (DOCX) [file pone.0157128.s009.docx]

S7 Table. Comparisons of *DNMT1* haplotype distributions in subgroups with the higher and lower levels of *DNMT1* mRNA expression.

| *DNMT1*  haplotype | Haplotype frequencies (N (ratio)) | | *p* | OR | 95% CI |
| --- | --- | --- | --- | --- | --- |
|  | Subgroup with Lower levels of *DNMT1* expression level | Subgroup with Higher Levels of *DNMT1* expression level |  |  |  |
| AGGT | 16 (0.098) | 9 (0.057) | 0.180 | 1.779 | 0.759 - 4.166 |
| GAAT | 37 (0.236) | 24 (0.154) | 0.068 | 1.691 | 0.958 - 2.984 |
| GGAT | 46 (0.290) | 57 (0.362) | 0.163 | 0.714 | 0.444 - 1.147 |
| AGAT | 11 (0.068) | 9 (0.056) | 0.680 | 1.212 | 0.485 - 3.033 |
| GGGC | 17 (0.105) | 25 (0.161) | 0.137 | 0.606 | 0.312 - 1.177 |
| GGGT | 22 (0.139) | 16 (0.101) | 0.304 | 1.433 | 0.720 - 2.850 |

The population was divided into subgroups with the lower and higher levels of *DNMT1* expression by the median of 2.37.

Loci for the haplotype analysis: rs2288349, rs2228611, rs8111085, and rs16999593.

N=158; *p* >0.05. (All those haplotype frequencies <0.03 will be ignored in analysis.)
