# Supplementary material for: Associations of ChREBP and Global DNA Methylation with Genetic and Environmental Factors in Chinese Healthy Adults
Source: PLoS One. 2016 Jun 9;11(6):e0157128. doi: 10.1371/journal.pone.0157128 (PMC4900669; doi:10.1371/journal.pone.0157128)
Supplement: S8 Table — (DOCX) [file pone.0157128.s010.docx]

**Table 8. Associations between *DNMT1* SNPs and lipid levels.**

| Loci | Model | FBG | TC | TG | HDL-C | LDL-C |
| --- | --- | --- | --- | --- | --- | --- |
| rs2288349 | additive | 0.917 | 0.890 | 0.769 | 0.950 | 0.755 |
|  | dominant | 0.519 | 0.524 | 0.831 | 0.472 | 0.995 |
|  | recessive | 0.275 | 0.089 | 0.762 | 0.176 | 0.432 |
| rs2228611 | additive | 0.396 | 0.776 | 0.214 | 0.122 | 0.668 |
|  | dominant | 0.419 | 0.761 | 0.164 | 0.198 | 0.126 |
|  | recessive | 0.606 | 0.905 | 0.674 | 0.129 | 0.124 |
| **rs8111085** | additive | 0.894 | 0.911 | 0.261 | 0.051 | 0.814 |
|  | dominant | 0.777 | 0.565 | 0.842 | 0.127 | 0.721 |
|  | recessive | 0.436 | 0.466 | **0.030** | 0.079 | 0.933 |
| **rs16999593** | additive | 0.879 | 0.092 | 0.648 | 0.184 | **0.039** |
|  | dominant | 0.922 | 0.064 | 0.661 | 0.269 | **0.023** |
|  | recessive | 0.829 | 0.849 | 0.814 | 0.250 | 0.824 |
| rs2336691 | additive | 0.728 | 0.673 | 0.591 | 0.680 | 0.417 |
|  | dominant | 0.728 | 0.673 | 0.591 | 0.680 | 0.417 |

FBG, fasting blood glucose concentration; TC, total cholesterol concentration; TG, total triglyceride concentration; HDL-C, high density lipoprotein cholesterol concentration; LDL-C, low density lipoprotein cholesterol concentration.

N=287; Adjusted for age, sex; Letters indicate the *p* values of associations in recessive, additive and dominant models between SNPs and lipid levels. Bold letters emphasize the *p* values < 0.05, indicating the significant SNP; All *p_FDR_* (the adjusted p for multiplying tests) > 0.05.
